# A Scoping Review of the Key Drivers That Impact Early-Career Nurses’ Thriving at Work, Intention to Stay in Employment, and Nursing Profession

**DOI:** 10.3390/nursrep16010002

**Published:** 2025-12-22

**Authors:** Hilda Masamba, Liz Ryan, Tracey Tulleners, Daniel Terry

**Affiliations:** 1 School of Nursing and Midwifery, University of Southern Queensland, Toowoomba, QLD 4350, Australia; liz.ryan@unisq.edu.au (L.R.); tracey.tulleners@unisq.edu.au (T.T.); daniel.terry@unisq.edu.au (D.T.); 2 Centre for Health Research, University of Southern Queensland, Springfield, QLD 4300, Australia; 3 Institute of Health and Wellbeing, Federation University Australia, Mt Helen, Ballarat, VIC 3353, Australia

**Keywords:** Early-Career Nurses, thriving, leadership support, organisational support, generational differences, work environment, work–life balance

## Abstract

**Background/Objectives:** The nursing profession is experiencing a global shortage of nurses. Early-Career Nurses (ECNs) assist in addressing the shortage; however, a significant number are leaving their workplaces and the profession. The aim of the review is to explore the factors that impact early-career nurses thriving at work, including their motivation and intention to stay in employment and the profession. **Methods:** A scoping review was conducted following the Joanna Briggs Institute (JBI) methodology. The objectives, analysis, and inclusion and exclusion criteria were informed by PRISMA for Scoping Reviews (PRISMA-ScR) to ensure accurate and complete reporting of findings. The target population were ECNs who are in the first five years of practice. Databases including CINAHL, PubMed, PsycINFO, Scopus, and Web of Science were searched to identify the literature on ECN thriving between 1985 and 2025. Titles, abstracts, and full texts of the identified studies were screened by two independent reviewers, and thematic analysis was undertaken to analyse the data. **Results:** A total of 190 studies was initially identified, and after screening and review, a total of 16 articles met the inclusion criteria and explored factors related to thriving and retention. Key themes identified within the literature that contribute to ECNs thriving at work encompass the work environment, work–life balance, and education, where generational differences may also create unique nuances between ECNs. **Conclusions:** ECNs encounter many challenges in the early stages of their nursing career. Organisational support may be responsive and provide conducive work environments that nurture growth, career development, and thriving for ECNs. However, future research is needed to further confirm drivers of thriving, along with understanding the impact of targeted interventions to better support ECN thriving and retention. Future search will include stakeholders to validate the findings.

## 1. Introduction

Early-Career Nurses (ECNs), defined as nurses within the first five years of practice, represent the future of the healthcare workforce [1,2]. High attrition rates among ECNs are contributing to global nursing shortages, placing additional strain on already overburdened healthcare systems [3]. Nursing shortages are projected to increase in the coming years [4,5], exacerbating the challenges faced by healthcare systems and compromising patient care delivery [6].

Poor staff retention and high attrition rates among nurses place significant pressure on healthcare services worldwide. Numerous studies have identified key reasons why nurses leave the profession, including high workloads, unfavourable patient-to-nurse ratios, burnout, and a lack of supportive leadership and mentorship [7,8,9,10].

While these issues affect nurses across all experience levels, ECNs are disproportionately impacted [1,3,11]. Many ECNs encounter transition shock, retention challenges [12], and a mismatch between generational expectations and workplace realities [13]. The early departure of ECNs from the workforce not only intensifies staffing shortages but also represents a loss of significant investment in training and education [14]. ECNs choose to leave the profession when they feel overwhelmed and unsupported [15,16].

Moloney et al. [8] identify key components that influence ECN retention and thriving in their workplace, where thriving is defined as a psychological state characterised by both vitality and learning, enabling individuals to grow, flourish, and feel energised, rather than merely ‘surviving’ in their roles [17]. Edmondson et al. [18] expand this definition to include physical wellbeing and performance, which are essential for professional development and success. This conceptualisation of thriving highlights the critical role of empowering leadership and organisational support in fostering ECNs’ professional growth and wellbeing [8]. Moloney et al. [8] highlighted nine critical areas of focus to address thriving, which include effective management, professional development, communication, scope of practice, autonomy, orientation, reward systems, and work–life balance.

While many healthcare services succeed in recruiting nurses, they are challenged in retaining and supporting ECNs to thrive [14]. Addressing these areas holistically at both individual and systemic levels can assist in mitigating attrition and promoting long-term engagement in the profession [19,20]. With the current nursing workforce spanning four generations, leveraging the unique strengths of each cohort offers a valuable opportunity to enhance ECN thriving [21]. Generational differences influence many aspects of nursing practice and should be thoughtfully integrated into strategic planning, support initiatives, and retention efforts [22]. Further research is needed to understand how these generational nuances impact ECN thriving and contribute to workforce sustainability.

Despite growing recognition of the challenges faced by ECNs, there remains a significant gap in the literature on generational diversity, organisational support, and thriving frameworks as important factors for retention. Although existing evidence acknowledges factors influencing ECNs’ motivation and intention to remain employed in the nursing profession, they often lack consistency in reporting, scalability, and longitudinal evaluation of interventions [16]. Sanches et al. [22] highlight the persistent challenges in adaptability and support, with limited interventions to address generational expectations or long-term career sustainability. These gaps in knowledge continue to limit the development of equitable strategies that support ECNs to not only stay but thrive in their nursing roles.

### Aim

To address this gap, the aim of the review is to explore the factors that impact early-career nurses thriving at work, including their motivation and intention to stay in employment and the profession.

## 2. Materials and Methods

A scoping review was conducted following the Joanna Briggs Institute (JBI) methodology for scoping reviews [23], utilising PRISMA for Scoping Review (PRISMA-ScR) guidelines to ensure accurate and complete reporting of findings [24].

### 2.1. Search Strategy

A search of the literature was undertaken between 7 August and 7 November 2024, with a follow up review on 23 September 2025, using PubMed, CINAHL, Scopus, Web of Science and PsycINFO databases using key search terms and strings, including ‘nurses’ OR nurs* OR ‘RN’ AND ‘early career’ OR Begin* OR ‘newly OR graduate* OR ‘junior’ OR transition to practice AND nurse residency OR ‘personnel turnover’ OR retain* OR stay* OR ‘leadership’ OR manage* OR supervis* OR ‘support* OR mentor* OR ‘leader’ AND ‘thrive’ OR ‘thrives’ OR ‘thriving’ OR prosper* OR flourish’ OR ‘career planning and development’ (Table A1). The focus was to capture the published literature regarding ECNs thriving at work and their intention to stay in their current job and the nursing profession. All of the literature published between 1985 and 2025 was searched to capture the evolution of evidence on early-career nurse transition and thriving. Although the conceptualisation of ‘thriving’ is relatively recent, earlier work (e.g., Duchscher’s seminal study on transition shock [25]) and related literature from the mid-1980s onward provide critical context for understanding factors influencing adaptation and retention. This broad time frame ensured the inclusion of foundational studies on transition and workforce sustainability alongside contemporary research on thriving [16,25]. Assistance was sought from the health research librarian to develop and refine the search strings and strategy. Hand searching and review of the reference lists of identified studies were also undertaken to identify any additional studies.

### 2.2. Inclusion and Exclusion Criteria

The review included original, peer-reviewed research as well as other relevant evidence sources (e.g., reviews, discussion papers) focused on ECNs working in any healthcare setting, including hospitals, community health, correctional facilities, Aboriginal and Torres Strait Islander Controlled Health Services, and general practice. The review encompassed any study published in English and excluded those focusing on nursing students or educational settings.

### 2.3. Study Screening and Extraction

**Figure 1:**
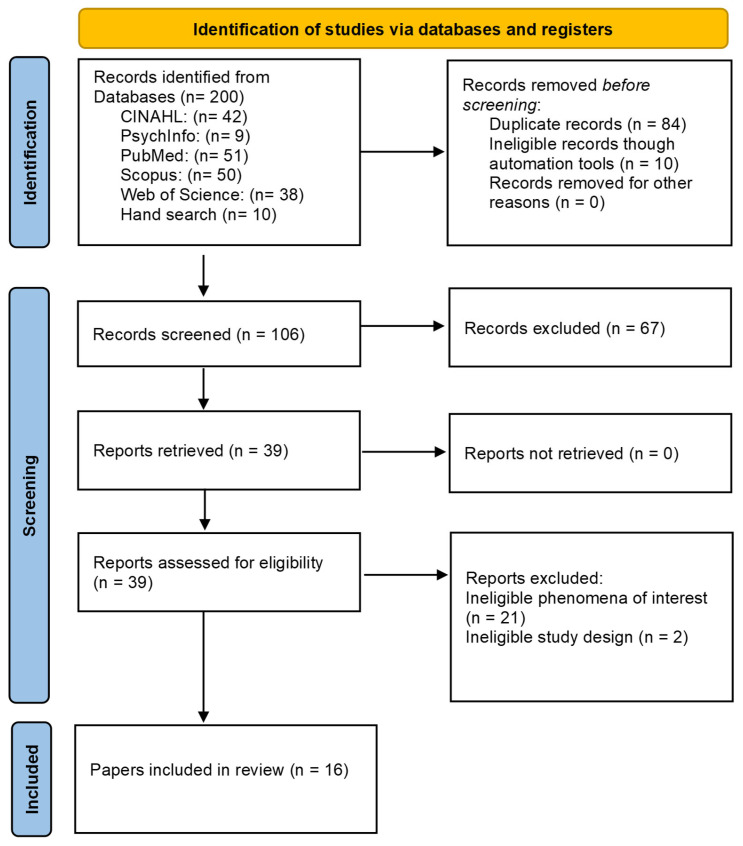
PRISMA-ScR flow chart.

All articles retrieved were exported and managed using JBI SUMARI software and EndNote (version 21). Initial screening was conducted by two reviewers (HM, DT), who removed duplicates and assessed titles, keywords, and abstracts to exclude irrelevant studies. The remaining full-text articles were then independently reviewed by two reviewers (HM, DT) and evaluated against the inclusion and exclusion criteria. Any studies classified as ‘not sure’ or where there was indecision between reviewers regarding inclusion were reviewed by a third reviewer (TT) and collectively discussed to reach a consensus (Figure 1).

### 2.4. Data Analysis

Data extraction was conducted using JBI SUMARI software. Thematic analysis was then used to identify, analyse, and report themes within the data [26]. Steps included becoming familiar with the data, generating initial codes, searching for themes, reviewing themes, defining and naming themes, and writing the report [26].

## 3. Results

A total of 200 records were identified through database searches. After removing duplicates and ineligible records (*n* = 94), 106 records remained. Title and abstract screening resulted in exclusions (*n* = 67). Full-text screening then led to further exclusions (*n* = 23). The final 16 identified articles spanned a range of methodologies, including case control, cohort, cross-sectional, qualitative, and systematic review designs and represent diverse healthcare settings across Australia (*n* = 3), New Zealand (*n* = 2), Canada (*n* = 3), the United States (*n* = 6), the United Kingdom (*n* = 1), and Japan (*n* = 1) (Table A2).

**Figure 2:**
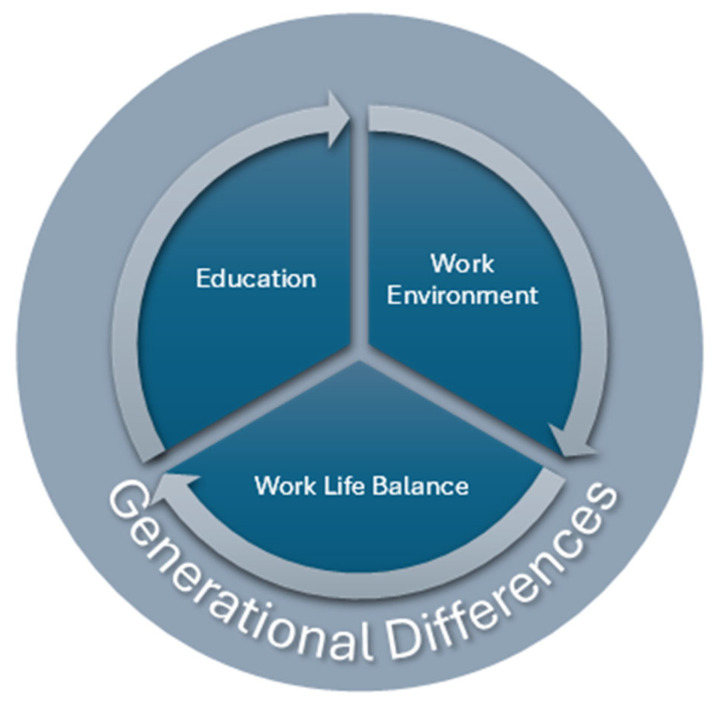
Key themes that impact ECN thriving.

Among the factors identified to contribute to ECNs thriving at work, these included the work environment, work–life balance, and education (Table A3). The following discussion provides details of these themes, noting that generational differences among the ECNs create unique nuances (Figure 2).

### 3.1. Work Environment

The work environment theme encapsulated all aspects of what ECNs observed and experienced at work. It comprises organisational, physical, and psychological factors that influence the nurse’s ability to thrive at work [27]. Satchell and Jacobs [10] reported poor work environments such as unmanageable workloads, inadequate staffing levels, and low pay as having the largest negative impact on ECNs’ ability to thrive. Additionally, unsupportive work environments and inadequate post-registration support affect retention and contribute to the attrition of ECNs [28]. D’Ambra and Andrews [29] stress the importance of the work environment for ECNs, evaluating how incivility influences new graduates’ transition experience.

New graduates were often expected to meet work expectations while feeling unwelcome, with little or no support, resources, or the ability to ask questions, resulting in ECNs leaving prior to completing orientation or within the first few months [30]. Abrupt and fragmented entry into nursing roles that did not recognise ECNs’ novice status affected their ability to thrive. Chandler [30] also highlighted that ECNs viewed some Certified Nursing Assistants as resources for information, while others were described as confrontational and unwilling to respond to questions. Harrison et al. [27] indicated that ECNs silently withdrew if they felt intimidated by senior or more experienced colleagues. Conversely, supportive work environments that promote teamwork, respect, acceptance, and a sense of belonging encourage ECNs to thrive in the workplace [27,30]. Other key elements of the work environment were central to ECNs feeling supported and are articulated through several sub-themes.

#### 3.1.1. Organisational Support

Beecroft et al. [31] report that organisations that value teamwork, cohesiveness, and collaboration are more likely to have committed employees. ECNs who were satisfied with their jobs and pay also felt committed to their organisation and thus had decreased turnover intention [31]. Such commitment was assisted through support from approachable nurse educators, well-prepared preceptors, and formal mentors, increasing ECNs’ practice readiness and job satisfaction [32].

In addition to supportive people, formal mentoring programmes, access to learning and development, and career advancement opportunities were vital for ECNs to engage and feel valued [31,33]. However, this required engagement by healthcare leaders to champion the professional development of ECNs [8]. ECNs expressed intentions to leave if they felt their learning and roles were stagnating [10]. High workloads, time constraints, and senior nurses being unavailable also impacted their ongoing learning [10].

Appropriate mentorship and structured programmes of support were indicated to prepare ECNs for the realities of nursing practice and equip them with emotional resilience for coping with the challenges of the nursing profession [29,31,32,33,34]. However, the complex nursing environment often made it more difficult to support the transition of ECNs due to external and internal political, organisational, economic, and regulatory factors [27].

#### 3.1.2. Empowering Leadership

In addition to organisational support, healthcare leaders play an important role in influencing the workplace environment and culture [28,34]. Boamah and Laschinger [35] report that leadership practices that promote organisational support and foster workplace conditions conducive to person–job fit are important for retaining newly graduated nurses. Likewise, leaders should be present and accessible to graduates. “Come out and show some interest in the ward. Don’t just come out and say, ‘good morning’ and then go back into your office” [28]. Africa and Harris [34] also highlight availability and unit presence where nurse managers participate in care delivery during high acuity periods. ECNs found their managers helpful when they were “able to help the flow of the unit and assist with patient care if the floor nurses are really busy with other patient care” [34].

Moloney et al. [8] identify leadership as practices that empower employees through shared power and decision-making. Their study highlighted strategies that created an environment where nurses were valued and given autonomy and opportunity for professional development and where leaders used effective and positive communication skills. Empowering leadership enabled nurses to thrive at work and was an important factor in reducing burnout and turnover intention [31]. Africa and Harris [34] provide insight into the nature of nurse manager and ECN relationships as requiring a foundational approach. Since ECNs are at the beginning of their nursing practice, this approach is based on effective communication and relationship building between nurse managers and ECNs.

ECNs appreciated leaders who were intentional and were there for them throughout their onboarding and transition experience, providing them time to adjust, with no overbearing demands [30]. “We don’t expect anything from you today, tomorrow or next week, just show up on time” [30]. Although Nurse Unit Managers were meant to be the key support people for new graduates, this was often difficult due to their other responsibilities [28]. Africa and Harris [34] proposed that nurse executives commit time and resources and take the lead in redesigning work systems that support nurse managers to increase nursing leadership engagement with ECNs.

#### 3.1.3. Interpersonal Relationships

Beyond empowering leadership, formal and informal interaction among nurses, such as colleagues and leaders, played a role in the integration of ECNs. ECNs are more committed to their organisation if interaction with others is encouraged to achieve individual and group goals [31]. Satchell and Jacobs [10] identified interpersonal relationships as an important factor in enabling thriving in the workplace. ECNs who were accepted by senior nurses felt that this was a powerful enabler and encouraged thriving in their role [36]. Further, ECNs who felt connected and accepted by their colleagues reported higher job satisfaction and a sense of belonging [28,29]. Fostering positive connections impacted learning, development, and the ability to concentrate on individual tasks and patient needs for ECNs [10].

In addition, ward culture influenced how ECNs integrated into the team [30]. Cultures described as competitive rather than collaborative did not welcome ECNs, who frequently felt like outsiders and received no support [30]. Healthcare leaders’ interactions with ECNs additionally influenced how other staff welcomed and supported them [27]. Leadership and management in the healthcare environment determine and influence workplace culture and how healthcare teams function, affecting ECN experiences [10,27].

In addition to leaders, peer interactions contributed to developing a sense of belonging and job satisfaction, where a lack of social peer support was associated with high turnover intention of ECNs [31]. ECNs supported each other, and this was a source of comfort for some, knowing that they were going through similar or shared experiences [30]. Additionally, day-to-day interactions and interpersonal relationships were highlighted as being impacted by coworker hostility. Incivility related to the perception that ECNs were lower in the nursing hierarchy, which impacted job satisfaction and workplace retention and, if seen as acceptable behaviour, impacted ECN job satisfaction and their commitment to the employer and profession [29]. The promotion of supportive relationships among coworkers is crucial to counter the effects of incivility. Collegial support significantly boosts ECN thriving and lowers their intention to leave the organisation [1].

#### 3.1.4. Incentives

In addition to interpersonal relationships, incentives were highlighted as a key contributor to ECNs thriving. Incentives included financial benefits, recognition, rewards, and promotion. ECNs also valued career development opportunities and indicated intention to leave their job where professional development opportunities were difficult to access [10]. They wished to be recognised, acknowledged, and rewarded for their contributions and wanted nurse managers and charge nurses to check in regularly to monitor their progress [30]. Remuneration packages were a key factor that influenced their perception of thriving [10]. Some nurses felt their wages did not equate to the level of responsibility and skill required, and having an increased income was associated with decreased turnover intention for ECNs [10].

Moloney et al. [8] highlight the importance of income incentives and rewards as a key component of organisational support for ECNs, noting their positive impact on vitality and engagement. Recognition of contributions, whether through financial compensation, paid time off, or other forms of reward, has been shown to enhance motivation and foster a more supportive work environment [35]. While such incentives may also correlate with reduced burnout, their primary value lies in reinforcing ECNs’ sense of purpose and professional growth. Boamah and Laschinger [35] recommend implementing structured reward systems and encouraging paid leave as part of a broader strategy to improve workplace conditions and reduce turnover intention. Supporting ECNs through meaningful incentives enables them to focus on gaining clinical experience, maintain wellbeing, and thrive in their roles without being overwhelmed by workplace pressures.

### 3.2. Work–Life Balance

Cole [37] further highlights the need for flexibility to help ECNs achieve work–life balance. Work–life balance encompasses ECNs seeking to manage their personal lives, while also managing their professional obligations within the transitional period of being a new nurse. Workplace stressors such as workload, redeployment, and coworker hostility can interact with life factors such as developmental assets and congruence and cause stress [37]. Stress in the workplace can carry over into personal life outside of work, thereby affecting work–life balance [32]. Thriving is significantly affected by workplace stressors that impact ECNs’ personal wellbeing and their new graduate role [38]. Therefore, achieving work–life balance enables thriving for ECNs [8].

The healthcare workspace is changing with more demands and challenges due to factors such as the aging population, more people experiencing chronic diseases, and increased patient acuity [36]. ECNs who are not well equipped for such challenges can become overwhelmed, and failure to cope can lead to stress and burnout [37]. Effective coping strategies may help to minimise stress and burnout in ECNs [8]. Burnout has a negative impact on the work–life balance of ECNs and causes psychological and physical symptoms [10]. Flexible work schedules and manageable workloads may allow ECNs time off to ‘recharge’ and manage personal commitments [38].

It is suggested that more experienced nurses are less likely to feel burdened by their workload due to their established proficiency [39], while ECNs focus on acquiring and building skills [30,32]. Harrison et al. [27] argued in favour of extending nurse training programmes to incorporate the first year of practice as part of the pre-registration programme to support graduate development and practice readiness.

Dames [38] noted that emotional readiness for professional practice was influenced by the development of congruence, self-esteem, and an ability to view workplace stimuli optimistically. However, it should be considered that no matter how an employee’s values are aligned with their role, workplace stressors have a significant impact on the ability to thrive [38]. Moloney et al. [8] highlighted the vulnerable mental health status of nurses following the global COVID-19 pandemic and advocated for organisations to support nurses by shortening shifts and allowing flexibility in shift schedules to promote rest hours and added work–life balance.

#### 3.2.1. Finding Positive Meaning

A sub-theme of work–life balance related to ECNs finding positive meaning in the work they were doing and the care they were providing. Some ECNs reported the provision of care to their patients as a meaningful experience, and this improved their daily job satisfaction and motivation to stay in the job [10]. Interference in an ECN’s ability to provide adequate care led to self-blame, stress, and burnout for some [10,35].

Several ECNs reported caring as central to their career identity and wanted to help and support people at their most vulnerable time [10]. However, most ECNs reported being overwhelmed by workloads as novice nurses [32]. ECNs who had undifferentiated workloads reported not being able to keep up with their more experienced colleagues and often missed breaks and finished late, suffering severe anxiety at work and in their personal lives [32]. Finding positive meaning was shown as a protective factor and improved their job satisfaction.

### 3.3. Education

Overall, ECNs were satisfied with their nursing education but felt they needed more clinical exposure and hands-on experience as students [30]. ECNs expressed the need to develop competency in skills and gain the confidence to be able to practice independently. Learning plays an important role in ECNs thriving in the workplace [10]. This is strengthened by access to learning opportunities that build competence, confidence, and adaptability [33]. Educational support combined with organisational support promotes the alignment of skill development with career aspirations [40].

Challenges were noted in rural settings where there was a lack of support post-graduation due to staff shortages, workloads not conducive to learning, and a lack of designated nursing staff for the transition to practice programme [28]. Supernumerary staffing arrangements were proposed to allow ECNs time to repeatedly practice skills and to attend professional development study days but were often unachievable due to rostering and staffing difficulties [28]. ECNs need to be accommodated, encouraged, and given time to learn new skills.

Harrison et al. [27] recommend the creation of nursing environments allowing graduate nurses to thrive and keep up with the contemporary complex healthcare contexts. They further stressed the role of organisational support in enabling nursing leaders to improve and build capacity in promoting a positive work culture [27]. Grindel [40] pointed out how the busy work environment was not supportive enough to nurture ECNs and how the lack of organisational commitment contributed to the cessation of mentorship programmes.

Mentors, nurse educators, and preceptors are influential in the growth and career development of ECNs and therefore should offer social support, encouraging engaging interactions that demonstrate value and respect for ECNs, allowing supported practice and learning [27,30,40].

### 3.4. Generational Differences

In addition to the themes currently identified in the literature, generational differences emerged. Although this theme was explicitly identified in only a small subset of studies (3 out of 16), it provides an important lens for understanding variations in ECN experiences. While not universally reported, the influence of generational expectations warrants consideration in workforce planning and support strategies. For example, Cole [37] highlights that Generation Y and Generation X ECNs differ significantly in their values, expectations, perceptions, and motivations, factors that are deeply relevant to how engaged nursing staff feel and how likely they are to remain in the workforce.

Supporting this, Nagai et al. [39] found that ECNs’ commitment and dedication are significantly enhanced by work environments that offer adequate professional resources. The study demonstrated that both personal and professional resources are key predictors of work engagement among ECNs. Given this, healthcare organisations must develop tailored strategies that not only meet the general needs of ECNs but also reflect the unique experiences and expectations of different generational cohorts. These strategies should be diverse, equitable, and inclusive [37]. Dames [32] noted that age interplayed with self-esteem and confidence, as well as promoting congruence. Acknowledging generational differences enables leaders to tailor support according to each generation’s needs to enable them to thrive. Growing up in different eras can shape ECNs’ work expectations and behaviour.

Cole [37] identifies key generational differences that shape how individuals engage with their work and make decisions about staying in their roles. Baby Boomers tend to be driven by achievement and value acknowledgment for their efforts, often demonstrating strong motivation, dedication, and collaboration. Generation X typically prioritises efficiency and work–life balance, showing a capacity for innovation and adaptability. Generation Y is motivated by meaningful work and a desire to make a positive impact, and they are likely to seek new employment if their expectations are not fulfilled. Generation Z, meanwhile, resists being confined to conventional workplace models and is more inclined to explore job opportunities frequently. As such, generational differences influence nearly every aspect of ECNs’ ability to thrive at work and must be acknowledged and integrated across all workforce planning, support, and retention strategies [37]. Exploration of intergenerational variations may help to inform workforce planning and the retention of ECNs.

## 4. Discussion

Emerging evidence indicates that the transition from student to ECN is a pivotal period marked by both opportunity and vulnerability. As ECNs navigate complex healthcare environments, their ability to thrive may be shaped by workplace culture, leadership, support systems, and generational dynamics [10,22,41]. The multifaceted factors influencing ECN integration, wellbeing, and retention are explored, highlighting the importance of relational leadership, structural supports, and inclusive practices [8,10].

Arrowsmith et al. [42] recognise the progression from student to ECN as a critical period often marked by stress, role ambiguity, and systemic gaps in support. While ECNs generally report satisfaction with their nursing education, many feel underprepared due to limited clinical exposure and hands-on experience during their studies [30]. This lack of practice readiness can adversely affect confidence and performance, contributing to reality shock, a phenomenon that impacts a significant number of nurses at the beginning of their careers and may result in missed care [43]. During transition, balancing personal responsibilities with the demands of a complex and often unforgiving healthcare environment presents a unique set of challenges among ECNs [40,44,45]. This may place additional pressure on novice nurses [8]. Work–life balance emerged as a critical factor in mitigating these pressures.

Flexible scheduling, adequate rest between shifts, and manageable workloads were suggested to be vital not only for physical recovery, but also for psychological wellbeing. The inability to disconnect from work can result in ECNs expressing feelings of isolation and distress, which highlights the need for rostering practices that are equitable, responsive, and supportive of recovery [32,46].

Person-centred graduate support models [47] and organisational strategies that prioritise excellence, kindness, and people-first values may be fundamental in creating environments where ECNs can thrive [48]. These approaches could include immediate feedback mechanisms, career counselling, and recognition of individual strengths and aspirations, including pathways beyond traditional hospital roles [49].

Work environment may have an influence on all aspects of nursing practice [8,10]. However, many healthcare settings remain fraught with persistent challenges such as inadequate staffing, high workloads, and limited support structures [50]. A healthy workplace, as defined by Leka et al. [51], encompasses physical, psychosocial, and organisational conditions that promote wellbeing and minimise harm. These ideals are not consistently realised in practice [52].

Systemic barriers and the unavailability of senior staff can restrict learning and development opportunities [10]. When ECNs perceive stagnation in their roles, intention to leave the profession increases [10,31]. Supportive relationships, particularly with nurse educators, preceptors, and mentors, remain critical to ECN development [10,27,29,30,31,32,33,34,37]. The absence of a unified framework for evaluating work environment factors can contribute to difficulty in prioritising strategies, and interventions risk being fragmented or misdirected [53]. Structural factors intersect with interpersonal dynamics in powerful ways, and ECNs who feel accepted and supported by colleagues report higher job satisfaction and a stronger sense of belonging [31,38]. Many of the same issues persist despite decades of research [30,32,38,40].

Leadership presence and support may be essential [28,34,35], but the discussion must go beyond acknowledging its value to interrogating how leadership is enacted and experienced in practice. A disconnect occurs between leadership ideals and everyday realities. For example, Nurse Unit Managers are positioned as key support figures for ECNs; however, their visibility and accessibility are often limited by competing responsibilities [28,32]. This highlights the structural constraints within healthcare organisations that may prevent leaders from achieving the supportive element within their roles, where leadership must be relational and engaged [54], not merely administrative [28].

Empowering leadership may address these gaps by promoting autonomy, shared decision-making, and valuing staff contributions, which aligns with contemporary understandings of workplace engagement and resilience, and its implementation requires a cultural shift [8,54]. The integration of compassionate leadership [55] further highlights that emotional intelligence and empathy are just as vital, challenging traditional hierarchies and promoting a reimagining of leadership as a collaborative and human-centred practice [56].

What has emerged is a tension between leadership as a formal role and leadership as a lived experience. ECNs do not respond to titles but to behaviours, where leaders are present, intentional, and responsive [30]. Moreover, the variability in leadership effectiveness across settings, particularly in rural or resource-constrained environments, suggests that a one-size-fits-all approach may be insufficient [57]. It is recommended that strategies should consider local context, workload distribution, and organisational culture. Without this nuance, leadership risks becoming performative rather than transformative [58,59]. Intentional investment in leadership development, structural support, and a shift toward relational models of care and management may be needed [8,34].

Incentives were reported to promote ECNs’ sense of thriving, and these extend beyond salary to include recognition, career development, and opportunities for advancement [8,10]. When ECNs are offered professional development and promotion pathways, they seem to interpret these as signs that their contributions are valued and their growth is supported [35,60].

Finding positive meaning in the act of caregiving was shown to be central to the wellbeing of ECNs [10,37]. However, when organisational stressors, such as poor staffing, incivility, and lack of support, interfere with the ability to provide quality care, this may lead to self-blame and emotional distress [30,35].

Cole [37] acknowledges how generational differences may influence ECN thriving. Fostering generational harmony may enhance team cohesion, reduce misunderstandings, and build trust, encouraging diverse perspectives and inclusive decision-making [41]. While this is particularly relevant in nursing, it also applies across sectors where generational dynamics impact workforce effectiveness [61].

### 4.1. Limitations

Key limitations of this study include the restricted capacity for in-depth critical appraisal since scoping reviews are designed to summarise existing evidence rather than evaluate it. This limits the ability to assess the strength of individual studies. Excluding non-English studies also limits cultural perspectives of ECNs from various backgrounds and how these impact ECN thriving in the nursing profession. Limited availability of papers discussing generational differences means that findings may not be generalisable to ECNs from different generations.

In addition, formal stakeholder consultation was not conducted as part of this review; however, the lead author is a stakeholder within the context of this research. Their professional experience informed the interpretation of the findings, but this does not replace a structured stakeholder engagement process. Future research should incorporate formal consultation to ensure broader perspectives and enhance the applicability of findings.

### 4.2. Implications for Practice

Thriving for ECNs is not an individual effort; whole organisational support is shown as a contributing enabler. Leaders were identified as playing a crucial role in influencing the work environment and organisational culture. In resource-limited areas—for example, rural settings—leaders had extra responsibilities for providing support to ECNs in a transition programme. Therefore, supporting leaders may enable them to provide holistic support for ECNs beyond their operational duties.

If available, deputy or associate nurse managers can be positioned to support nurse managers; this may allow them more time to provide guidance to ECNs. In addition, support can include training to equip leaders with tools such as transformational leadership, awareness of ECNs’ needs for thriving at work, and being available and approachable to foster an inclusive culture that unites all generations in the nursing workforce today.

Further, all ECNs should feel safe in their work environment and be recognised as valuable members of the team. Mentors can play an important role in orienting and supporting ECNs during the transition into nursing and assisting in rapport building and maintaining relationships with a multidisciplinary team. Lastly, there is a need to investigate the allocation of workloads to ECNs, especially in the transition period, while future healthcare policies should consider specific ECN thriving strategies in workforce planning and retention. The implications identified in this review should be interpreted as suggestions, given the limited evidence base of scoping reviews. Therefore, future research may help to confirm these drivers, explore their impact, and evaluate the effectiveness of targeted interventions for early-career nurse thriving and retention.

## 5. Conclusions

Key drivers impacting ECN thriving and their motivation and intention to stay in their current jobs and the nursing profession are mapped in this scoping review. Findings indicate that ECNs face multiple challenges in the early years of their career, exacerbated by increasingly demanding healthcare environments and workforce shortages. ECN vulnerability in the transition period needs to be acknowledged and appropriate interventions implemented. Evidence suggests that organisational responses that include targeted strategies for ECN thriving are crucial. Empowering leadership, organisational support, and inclusive work cultures should strive to foster conditions that nurture career development, belonging, and wellbeing, as reported in this review. ECN thriving is shown as an important factor for workforce retention to mitigate future problems of early attrition and deficits in the nursing profession. It is suggested that healthcare leaders also require support for them to effectively influence a work environment culture that enables ECN thriving. Work environment, work–life balance, education, and generational factors may play a part in promoting a thriving nursing workforce where ECNs can flourish, as indicated in the literature. Further research may contribute to a greater understanding of a nursing workforce that enables thriving and sustaining ECNs. Challenges were noted that are not only unique to ECNs. Other professions also experience similar challenges in the early years of their careers and need strategic support. Embracing generational differences may promote the nurturing of future nursing leaders who are responsive to building a resilient nursing workforce that is centred on belonging.

## Data Availability

All data supporting the findings of this nursing report are publicly available online. References and supplementary materials can be accessed via their respective DOIs or URLs, as listed in the References section.

## Public Involvement Statement

No public involvement in any aspect of this research.

## Guidelines and Standards Statement

This manuscript was drafted against the PRISMA Extension (PRISMA-ScR) [24] for Scoping Reviews.

## Appendix A

**Table A1:** Search Terms.

Database	Search Terms
PubMed	(“nurses” OR nurs* OR “RN”) AND (“early career” OR Begin* OR “newly” OR graduate* OR “junior” OR “transition to practice”) AND (“nurse residency” OR “personnel turnover” OR retain* OR stay*) AND (“leadership” OR manage* OR supervis* OR support* OR mentor* OR leader) AND (“thrive” OR thrives OR thriving OR prosper* OR flourish OR “career planning and development”)
CINAHL	(“nurses” OR nurs* OR “RN”) AND (“early career” OR Begin* OR “newly” OR graduate* OR “junior” OR “transition to practice”) AND (“nurse residency” OR “personnel turnover” OR retain* OR stay*) AND (“leadership” OR manage* OR supervis* OR support* OR mentor* OR leader) AND (“thrive” OR thrives OR thriving OR prosper* OR flourish OR “career planning and development”)
Scopus	TITLE-ABS-KEY((“nurses” OR nurs* OR “RN”) AND (“early career” OR Begin* OR “newly” OR graduate* OR “junior” OR “transition to practice”) AND (“nurse residency” OR “personnel turnover” OR retain* OR stay*) AND (“leadership” OR manage* OR supervis* OR support* OR mentor* OR leader) AND (“thrive” OR thrives OR thriving OR prosper* OR flourish OR “career planning and development”))
Web of Science	TS = (“nurses” OR nurs* OR “RN”) AND TS = (“early career” OR Begin* OR “newly” OR graduate* OR “junior” OR “transition to practice”) AND TS = (“nurse residency” OR “personnel turnover” OR retain* OR stay*) AND TS = (“leadership” OR manage* OR supervis* OR support* OR mentor* OR leader) AND TS = (“thrive” OR thrives OR thriving OR prosper* OR flourish OR “career planning and development”)
PsycINFO	(“nurses” OR nurs* OR “RN”) AND (“early career” OR Begin* OR “newly” OR graduate* OR “junior” OR “transition to practice”) AND (“nurse residency” OR “personnel turnover” OR retain* OR stay*) AND (“leadership” OR manage* OR supervis* OR support* OR mentor* OR leader) AND (“thrive” OR thrives OR thriving OR prosper* OR flourish OR “career planning and development”)

**Table A2:** The 16 identified papers and factors that affect thriving.

Author [Reference]	Country	Design	Health Service Setting	Results
Africa and Harris [32]	US	Cross-sectional survey	Healthcare organisations across 4 states (14 different department types)	Performance evaluation by managers found to be helpful by ECNs Availability of managers and unit presence enables ECN thriving ECNs valued provision of information and support from managers Timely communication and follow-through benefited ECNs
Beecroft et al. [31]	US	Quantitative prospective cohort study	Paediatric hospitals	Supportive organisations reduce intent to leave Increased support-seeking linked to turnover risk Job satisfaction and pay reduced turnover intention for ECNs Positive work environments reduce turnover intention Older ECNs indicated turnover intent if first choice of ward not received
Boamah and Laschinger [38]	Canada	Quantitative cross-sectional survey	Acute setting	A better work–life fit reduces burnout Workplace support is crucial for improving work–life fit and reduces interference Organisational management should implement strategies to enhance workplace conditions that promote person–job fit and work–life balance to improve nurse retention
Chandler [30]	US	Qualitative study using appreciative inquiry	Various (sites not specified)	Formal orientation programmes enable ECN thriving Transition is challenging for ECNs Good communication with leaders and colleagues helped ECNs feel safe to ask questions Supportive relationships with approachable managers and mentors enable ECN thriving Feedback and reflection enhance learning and development of confidence
Cole [37]	UK	Literature review	n/a	Work–life balance is important to ECNs Generational differences affect all areas of ECN thriving Organisational culture and support Incentives and recognition contribute to ECN thriving
D’Ambra and Andrews [29]	US	Integrated literature review	n/a	Incivility is a major stressor, particularly for ECNs Low job satisfaction is associated with incivility Mentors are critical supports for ECNs facing incivility Nurse leaders need to create supportive work environments and culture Organisations need to implement strategies to eliminate incivility instead of promoting tolerance or overlooking uncivil behaviours in the nursing environment
Dames [36]	Canada	Qualitative study	City hospital, community, and large city hospital	Interaction between workplace conditions and developmental assets influences thriving Some ECNs thrive while others struggle in similar environments, therefore individualised support required to ensure thriving of all ECNs Nurse leaders need to address both environmental stressors and personal development to support ECN thriving
Dames [39]	Canada	Qualitative study	City hospital, community, and large city hospital	The ability to manage workplace challenges is linked to emotional resilience ECNs who lack developmental assets are likely to perceive workplace stimuli as threatening, which hinders thriving Transition programmes need to address work environment and development needs of ECNs
Grindel [35]	US	Professional commentary/report	n/a	Formal mentorship programmes helped to reduce ECN turnover and workforce challenges Mentorship improves ECN confidence and competence Organisational commitment and support help the integration of ECNs
Harrison et al. [27]	Australia	Qualitative case study (grounded theory)	Four healthcare provider institutions	Supportive relationships with leaders, mentors, and peers are important for ECN thriving Workplace context influences practice readiness and thriving Leaders’ interactions with ECNs influence how they are supported Multidisciplinary approach helps to prepare ECNs to thrive in the transition period
Lea and Cruickshank [28]	Australia	Qualitative case study	Rural practice	Visibility of leaders and role clarity for ECN mentors during transition Challenges exist in rural areas where managers also serve as the main support for ECNs Leaders should provide emotional support and create a culture that comprises ECN development Leaders need to encourage professional development opportunities for ECNs Support required for leaders to enable them to support ECNs
Moloney et al. [8]	NZ	Mixed methods sequential explanatory study	Primary healthcare	Thriving at work is mainly influenced by empowering leadership and organisational support Thriving reduces turnover intention Vitality was linked to increased wellbeing and reduced intention to leave organisations and profession Peer support is positively associated with thriving
Nagai et al. [42]	Japan	Quantitative longitudinal study	7 general hospitals 4 rural areas 3 urban	Organisational factors such as leadership, teamwork, and development opportunities were critical in sustaining engagement ECNs benefit from tailored support Thriving work environments reduce turnover
Satchell and Jacobs [10]	NZ	Qualitative descriptive study	Large tertiary hospital	Work environment influences ECN thriving and experiences Professional development improves growth and thriving ECNs find positive meaning in the act of caregiving Interpersonal relationships and interactions with colleagues and managers were central to thriving ECN thriving is influenced by various factors and requires systemic support
Terry et al. [1]	Australia	Quantitative cross-sectional study	Various (sites not specified)	Positive collegial relationships are associated with higher engagement Fostering strong collegial relationships and support systems is important for retaining nurses and enhancing wellbeing Positive relationships contribute to thriving at work Both individuals and organisations are responsible for creating an environment where nurses can thrive
Young and Ball [33]	US	Discussion and implementation commentary	Ambulatory Care	Recruitment and retention of ECN are improved by formal residency programmes ECN thriving is improved by organisational support and leadership involvement Supportive onboarding experience and mentorship involvement improve ECN thriving

**Table A3:** Key themes identified.

Author [Reference]	Work Environment	Work–Life Balance	Education	Generational Differences
Africa and Harris [32]	Y	Y	Y	-
Beecroft et al. [31]	Y	Y	-	-
Boamah and Laschinger [38]	Y	Y	-	-
Chandler [30]	Y	-	Y	-
Cole [37]	Y	Y	-	Y
D’Ambra and Andrews [29]	Y	-	Y	-
Dames [36]	Y	Y	-	-
Dames [39]	Y	Y	-	Y
Grindel [35]	Y	-	Y	-
Harrison et al. [27]	Y	-	Y	-
Lea and Cruickshank [28]	Y	-	Y	-
Moloney et al. [8]	Y	Y	-	-
Nagai et al. [42]	Y	Y	-	Y
Satchell and Jacobs [10]	Y	Y	-	-
Terry et al. [1]	Y	Y	Y	-
Young and Ball [33]	Y	-	Y	-
Y = Yes, includes theme				
